# Effectiveness of nitrogen fixation in rhizobia

**DOI:** 10.1111/1751-7915.13517

**Published:** 2019-12-04

**Authors:** Kristina Lindström, Seyed Abdollah Mousavi

**Affiliations:** ^1^ Faculty of Biological and Environmental Sciences and Helsinki Institute of Sustainability Science (HELSUS) University of Helsinki FI‐00014 Helsinki Finland

## Abstract

Biological nitrogen fixation in rhizobia occurs primarily in root or stem nodules and is induced by the bacteria present in legume plants. This symbiotic process has fascinated researchers for over a century, and the positive effects of legumes on soils and their food and feed value have been recognized for thousands of years. Symbiotic nitrogen fixation uses solar energy to reduce the inert N_2_ gas to ammonia at normal temperature and pressure, and is thus today, especially, important for sustainable food production. Increased productivity through improved effectiveness of the process is seen as a major research and development goal. The interaction between rhizobia and their legume hosts has thus been dissected at agronomic, plant physiological, microbiological and molecular levels to produce ample information about processes involved, but identification of major bottlenecks regarding efficiency of nitrogen fixation has proven to be complex. We review processes and results that contributed to the current understanding of this fascinating system, with focus on effectiveness of nitrogen fixation in rhizobia.

## Graphical abstract

The effectiveness of rhizobia in symbiosis with legumes is reviewed, with references from older and most recent literature.

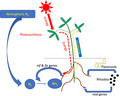

## Efficiency of rhizobia–legume symbiosis

Nitrogen is an essential component of urea and amino acids (proteins), nucleic acids (DNA and RNA), adenosine triphosphate (ATP) and nicotinamide adenine dinucleotide (NAD) in all living cells (Burén and Rubio, 2017). Nitrogen is a major component of chlorophyll, the most important pigment required for photosynthesis, and plays a critical role for plant growth and production (Wagner, 2012). Dinitrogen (N_2_) is the most available gas and the major constituent of the atmosphere. However, only the reactive types of nitrogen like oxidized (e.g. NO_x_) or reduced (e.g. NH_3_ and amines) nitrogen types can be assimilated by plants (Burén and Rubio, 2017). The conversion of N_2_ to the biologically available form of nitrogen (NH_3_) could be performed either by the industrial Haber‐Bosch process or via biological nitrogen fixation by certain bacteria and archaea (diazotrophic prokaryotes).

Rhizobia is a generic name for a certain Gram‐negative group of *Alphaproteobacteria* and *Betaproteobacteria* that can form nodules on the root, or in some cases on the stems, of their hosts and fix nitrogen in symbiosis with legumes as their host plants (Garrity *et al.*, 2005; Sprent, 2008). The genus *Rhizobium* was the first described group of these bacteria, and that is why this name has been frequently used for the nitrogen‐fixing bacteria of legumes. Today, this group of bacteria are scattered in 18 genera in the families *Rhizobiaceae* (*Rhizobium*, *Ensifer (*syn. *Sinorhizobium)*, *Allorhizobium*, *Pararhizobium*, *Neorhizobium, Shinella), Phyllobacteriaceae* (*Mesorhizobium, Aminobacter, Phyllobacterium), Brucellaceae* (*Ochrobactrum), Methylobacteriaceae* (*Methylobacterium, Microvirga), Bradyrhizobiaceae* (*Bradyrhizobium), Xanthobacteraceae* (*Azorhizobium), Hyphomicrobiaceae* (*Devosia)* and *Burkholderiaceae* (*Paraburkholderia*, *Cupriavidus*)*.* The genus *Rhizobium,* accommodating 112 species, is the largest genus of rhizobia (Mousavi, 2016; de Lajudie *et al.*, 2019; http://www.bacterio.net/).

The recent history of the symbiotic nitrogen fixation process offers a fascinating tour of the development of this branch of biological research. The so‐called ‘energy crisis’ in the late 1970s, a consequence of raising oil prices on the world market, sparked a renewed interest in the mechanisms behind the process, which uses solar energy to drive the production of combined nitrogen for plant growth. In this review, we start at that point and proceed until the current climate and environmental crises. We search for factors contributing to the efficiency or effectiveness of the process, a goal that is as important today as in the 1970s, when trying to feed the world population in a sustainable way.

We also chose to include work on which current advances build upon. Before the molecular and technical advances that we now take for given, careful and sophisticated experiments were designed, performed and reported, building the foundations for today’s knowledge step by step. For this part, we mainly rely on review articles written by experts of their time.

The words efficiency, efficacy and effectiveness are used in rhizobium literature almost without distinction. However, since they do mean different things, a distinction might be useful when discussing symbiotic nitrogen fixation (SNF) in associations between rhizobia and legumes. According to encyclopaedia (e.g. Wikipedia, www.wikipedia.org and Diffen, https://www.diffen.com), **efficiency** means how well something is done. In biological systems, efficiency is defined as the effectiveness with which energy is used for a particular process. Energy efficiency is calculated by relating energy use to minimum energy cost under defined conditions (Phillips, 1980). For rhizobial SNF, this would mean the function of the nitrogenase enzyme: energy use in SNF and the outcome of the process in terms of N_2_ fixed. **Efficacy** defines how well a system or product functions under ideal conditions; in rhizobium terms, this could mean how much nitrogen is fixed, for example, in axenic culture in pouches, test tubes or enclosed jars, taking the whole system into account. However, this term is seldom used in the context. **Effectiveness** again indicates how useful the system is in practice. For the legume–rhizobium symbiosis, this term could mean how productive the system is for our purposes, i.e. dry matter and protein production.

In SNF research, efficiency and effectiveness of nitrogen fixation are often used in parallel. Strictly speaking, efficiency should be used when we take an energy perspective, taking into account the function of the nitrogenase enzyme as well as energy provision by the plant to support the reaction. Effectiveness would again mean the outcome of the whole system, including in addition to energy use, all other factors contributing to a successful symbiosis.

**Community‐level efficiency** was introduced by Phillips (1980) to stand for the amount of N_2_ reduced per unit of land area. This expression is hardly longer in use. However, it shows how much emphasis was put on the energy aspect in the 1970s and 1980s when the so‐called energy crisis prompted the society to look for alternative, sustainable means of energy production to replace fossil fuels. The attention was turned to biological nitrogen fixation, which uses biological processes for transformation of atmospheric N_2_ gas to biologically useful ammonia, in contrast to the Haber‐Bosch method, which relies on fossil energy.

The research on SNF has through the years taken diverse routes to reach the common aim of replacing synthetic N fertilizer with N reduced by the enzyme nitrogenase. Today, one line of research aims at applying synthetic biology and biotechnology to engineer a biocatalyst for fertilizer production. Another main direction is to take on the challenge of engineering non‐legumes to either harbour nitrogenase without rhizobial infection or to become nodulated by rhizobia. Studies with these aims result in a great deal of basic research on biological mechanisms of SNF, some of which could be applied when looking for efficiency and trying to understand effectiveness in rhizobia. Two different concepts should be kept in mind when dealing with this topic, namely nodulation efficiency and N_2_‐fixation efficiency. This minireview mainly deals with the latter, though it is not wise to draw sharp borderlines between concepts defined to help us keep track on complex biological processes.

## Rhizobia and SNF

**Figure 1:**
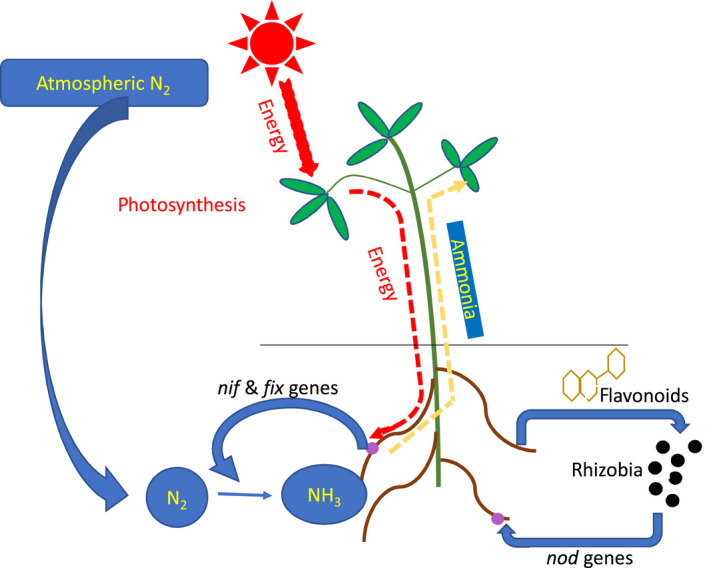
Summarized model for symbiotic nitrogen fixation in legumes by rhizobia.

The best‐known group of symbiotic nitrogen‐fixing bacteria are the rhizobia. However, two other groups of bacteria including *Frankia* and *Cyanobacteria* can also fix nitrogen in symbiosis with plants. Rhizobia fix nitrogen in plant species of the family *Leguminosae*, and species of another family, e.g. *Parasponia*. *Frankia,* can nodulate actinorhizal plants and *Cyanobacteria* can enter a symbiotic relation with a wide range of plants (Dawson, 2008; Normand and Fernandez, 2009; Lindström and Mousavi, 2010; Op den Camp, *et al.*, 2011; Mousavi, 2016), according to the definition ‘Symbiosis is the acquisition of an organism(s) by another unlike organism(s), and through subsequent long‐term integration, new structures and metabolism(s) emerge’ (Zook, 2015). The SNF interaction between rhizobia and legumes is a mutual symbiosis in which both plants and bacteria are benefited. In this symbiotic relationship, rhizobia are hosted and supplied with carbon sources by legumes and in return legumes receive ammonia provided by rhizobia (Fig. 1).

Symbiotic nitrogen fixation in rhizobia is mostly controlled by *nod*, *nif* and *fix* genes (Table 1). Nod and Nif proteins are encoded by accessory genes of bacteria that are placed in transmissible genetic elements like plasmids, symbiotic islands and chromids. Thus, these sets can be transferred horizontally in high frequencies within the species of a bacterial genus and infrequently between genera (Remigi *et al.*, 2016). The rhizobial lipo‐chito‐oligosaccharide signal molecules called Nod factors are encoded by a unique set of genes of rhizobia called *nod* genes. They are important genes for bacterial invasion and induction of nodules in the early stages of nodulation by inducing host responses such as root hair deformation and cortical cell division (Moulin *et al.*, 2004). Recently, the role of rhizobial NFs has been revisited, as their interaction with NF receptors and chitinases point to a putative role in the balance between symbiosis and defence (Kelly *et al.*, 2017). The interaction between legume roots and rhizobia is initiated by the exudation of flavonoids by legumes that is recognized by rhizobial NodD, which in turn regulates the transcription of other nodulation genes (*nod*, *nol*, and *noe*) in rhizobia (Bonaldi *et al.*, 2010; Rogel *et al.*, 2011). The three *nod* genes, *nodA, nodB* and *nodC,* exist as single copy genes in most rhizobia. They are involved in synthesis of the Nod factor backbone (Moulin *et al.*, 2004). According to Bonaldi *et al., *(2010), three *nod* genes are essential for the synthesis of the NF core structure: i) *nodC* encodes an N‐acetyl‐glucosaminyltransferase that polymerizes UDP‐N‐acetyl‐D‐glucosamine into oligosaccharide chains, ii) *nodB* encodes a deacetylase that removes the N‐acetyl moiety from the non‐reducing terminus of these oligosaccharides, and iii) *nodA* encodes an acyltransferase that links an acyl chain to the non‐reducing end of the oligosaccharides. Moreover, the different other nodulation genes (*nod*, *nol* or *noe*) can be found in different rhizobial species, being more or less essential for the host specificity of each species, by decoration of the basic Nod factor backbone, leading to different substitutions, such as acetylation, glycosylation, methylation and sulfation (Bonaldi *et al.*, 2010; Andrews and Andrews, 2017). It is worth noting that Giraud *et al. *(2007) reported that two symbiotic, photosynthetic, *Bradyrhizobium* strains, BTAi1 and ORS278, can establish nodules on stem and roots of *Aeschynomene indica* and *A. sensitiva* in the absence of *nodABC* and lipochito‐oligosaccharidic Nod factors (NF). This phenomenon was also found in a symbiotic relationship between a NF‐independent mutant of *B. elkanii* and *Glycine max* cv. Enrei, as well as the symbiosis between the model plant *Lotus japonicus* and NF mutants of *Mesorhizobium loti* (Madsen *et al.*, 2010; Okazaki *et al.*, 2013). For NF‐independent symbiosis, Okazaki *et al. *(2016) proposed a process dependent on the type III secretion system (T3SS) by which rhizobial effectors are translocated to certain legume species to activate symbiosis in host legumes. Recognition of NFs by legumes is mediated by Nod factor receptors (NFRs) that are plasma membrane localized serine/threonine receptor kinases that contain sugar‐binding LysM domains. NFR1 and NFR5, and NFP and LYK3 are the reported NFRs in the case of the model legumes *Lotus japonicus* and *Medicago truncatula,* respectively (Madsen *et al.*, 2003; Andrews and Andrews, 2017).

**Table 1:** A list of the most common rhizobial *nod*, *nif* and *fix* genes (after Laranjo *et al.*, 2014).

Genes	Function of gene product
Nodulation genes	
*nodA*	Acyltransferase
*nodB*	Chitooligosaccharide deacetylase
*nodC*	N‐acetylglucosaminyltransferase
*nodD*	Transcriptional regulator of common *nod* genes
*nodIJ*	Nod factor transport
*nodPQ*	Synthesis of Nod factor substituents
*nodX*	Synthesis of Nod factor substituents
*nofEF*	Synthesis of Nod factor substituents
Other *nod* genes	Several functions in synthesis of Nod factors
*nol* genes	Several functions in synthesis of Nod factor substituents and secretion
*noe* genes	Synthesis of Nod factos substituents
Nitrogen fixation genes	
*nifH*	Dinitrogenase reductase (Fe protein)
*nifD*	α subunits of dinitrogenase (MoFe protein)
*nifK*	β subunits of dinitrogenase (MoFe protein)
*nifA*	Transcriptional regulator of the other *nif* genes
*nifBEN*	Biosynthesis of the Fe‐Mo cofactor
*fixABCX*	Electron transport chain to nitrogenase
*fixNOPQ*	Cytochrome oxidase
*fixLJ*	Transcriptional regulators
*fixK*	Transcriptional regulator
*fixGHIS*	Copper uptake and metabolism
*fdxN*	Ferredoxin

Colonization of legume roots by rhizobia results in the deformation and curling of root hairs, and several genes, e.g. early nodulation (*ENOD*) genes and *Msrip1*, are expressed in the epidermis of legumes (Franssen *et al.*, 1995; McAdam *et al.*, 2018). Several recent reviews deal with the complex biological interactions leading to infection and nodulation by rhizobia, such as how the legume controls the massive infection caused by rhizobia (Berrabah *et al.*, 2018; Buhian and Bensmihen, 2018; Poole *et al.*, 2018). The two major types of nodules formed are classified as indeterminate or determinate. Indeterminate nodules are characterized by maintaining meristematic tissue, whereas determinate nodules are spherical and have a transient meristem. The type nodule is determined by the host plant and in rare cases legumes may form both nodule types (Andrews and Andrews, 2017).

The Nif family of rhizobia plays a crucial role in SNF by encoding the nitrogenase complex as well as a number of regulatory proteins involved in nitrogen fixation. *Nif* genes can be found in other groups of bacteria than rhizobia as well. Besides *nif* genes, regulation of nitrogen fixation is dependent on the so‐called *fix* genes. The two main regulatory cascades that can be found in rhizobia are the RpoN‐NifA and the oxygen‐responsive two‐component FixL‐FixJ system, together with FixK. NifA is the master regulator of nitrogen fixation and belongs to the enhancer‐binding protein family of transcriptional regulators that interact with RNA polymerase sigma factor, σ54 (RpoN). The group of Hauke Henneke greatly contributed to the early exploration of *nif* and *fix* genes in *Bradyrhizobium japonicum* (e.g. Fischer and Hennecke, 1984; Fischer *et al.*, 1986; Fischer, 1994). In the case of *Rhizobium etli* CFN42, *rpoN* is expressed in two copies: RpoN1 and RpoN2 (Girard *et al.*, 2000; Dixon and Kahn, 2004; Salazar *et al.*, 2010). Rpon1 is encoded in the chromosome in free‐living conditions, whereas *rpoN2* is located in the symbiotic plasmid and is expressed independently of *rpoN1* in bacteroids (Michiels *et al.*, 1998; Salazar *et al.*, 2010). Österman *et al. *(2015) showed that *Neorhizobium galegae* sv. orientalis required *rpoN2* for nitrogen fixation. Interestingly, *rpoN2* could be found only in symbiovar orientalis but not in the other symbiovars, *N. galegae* sv. officinalis. The other regulator cascade (FixL‐FixJ‐FixK) is one of the two regulators of transcription of *nifA* and *fix* genes in SNF (Dixon and Kahn, 2004).

## Energy aspects of nitrogenase

The nitrogenase enzyme complex is the central unit of nitrogen fixation in all known diazotrophic organisms, which are all prokaryotes. The development of biochemistry opened up avenues to characterize and understand nitrogenase and SNF. Non‐symbiotic N_2_‐fixing bacteria were better amenable to studies of the structure and function of nitrogenase. The first report on the activity of nitrogenase enzyme preparations from *Clostridium pasteurianum* was by Carnahan *et al. *(1960). Bulen and LeComte (1966) in their paper *The nitrogenase system from Azotobacter: two‐enzyme requirement for N_2_ reduction, ATP‐dependent H_2_ evolution, and ATP hydrolysis*, described the function of dinitrogenase of *Azotobacter vinelandii, Clostridium pasteurianum* and *Rhodospirillum rubrum*, paving the path for further study into this complex system. Mortenson and Thorneley (1979) in their review described in detail the advances of the knowledge of the two‐component nitrogenase enzyme system–the Fe protein, a dimer, and the tetrameric FeMo‐Co protein with its cofactor.

Nitrogenase from the rhizobium–legume symbiosis was isolated by Whiting and Dilworth (1974), while there was still uncertainty about the role of the bacteria vs. the host plant regarding the location of the nitrogenase‐encoding *nif* genes. However, based on genetic mapping of *Klebsiella* and *Rhizobium*, already Streicher *et al., *(1972) speculated about the construction of rhizobial strains with high effectiveness and broad host range, based on transformation of strains, and Hardy and Havelka (1975) outlined a scheme for the transfer of *nif* genes to plants. The transfer to other bacteria became a realistic alternative with the detection of *nif* gene carrying plasmids in *Rhizobium leguminosarum* (Nuti *et al.*, 1979), but so far, no transfer of functional *nif* genes to plants has been reported. The construction of the broad host range cloning system by Ditta *et al. *(1980) opened up the door for more precise characterization of *nif* and other symbiotic genes. This line of molecular research, making use of the new powerful tools, soon became mainstream.

Before the molecular revolution, however, research on efficiency went through interesting and important stages. Minchin and Pate (1973) performed detailed studies on the carbon balance of the legume and the functional economy of its root nodules, using the garden pea (*Pisum sativum*) inoculated with *Rhizobium* strain V 200 as their model legume. Their studies relied on thorough studies on legume physiology reported in a series of papers from Dr Pate’s group, by adding the perspective of energy economy to the functioning of the symbiotic system. They found that the respiratory efficiency of a nodulated root in terms of nitrogen fixation was very similar to that of an uninoculated root assimilated nitrate, i.e. 5.9 vs 6.2 mg C/mg N fixed or assimilated. A mean value of 6 mg C/mg N was obtained by Vance and Heichel (1991) over 35 determinations. Phillips (1980) in his extensive review compiled information from a range of studies on the whole‐plant energy costs of symbiotic N_2_ fixation in nodulated legumes. The estimated costs expressed as gC/gN varied from below 1 to 20, depending on the method used for the estimation of N_2_ fixation. Values around 6 gC/gN were obtained when the acetylene reduction method was used to measure nitrogenase activity. Other methods used were the N difference method, obtained by comparison of dry matter and N yields in nitrogen‐fixing and a non‐nodulated crop, or CO_2_ evolution in relation to N content of nodules or nodulated roots or alternatively, ^15^N gas incorporated into these.

Estimation of the amounts of N fixed is crucial in all kinds of N_2_ fixation research. It is also a question of much debate. Herridge *et al. *(2008) discussed several methods, which were further listed in Lindström and Mousavi (2010): (1) Acetylene reduction, which is an indirect method mainly useful for nitrogenase activity comparisons; (2) total nitrogen balance or determination of nitrogen inputs and outputs in soil and crops; (3) nitrogen difference, obtained by comparing a nitrogen‐fixing crop with a suitable non‐fixing crop; (4) ^15^N isotope accumulation (incubation with heavy isotope‐enriched nitrogen gas); (5) ^15^N isotope dilution (application of ^15^N enriched N fertilizer, which is taken up by mainly non‐fixing plants) and (6) natural ^15^N isotope abundance. The nitrogen difference method is the simplest and most straightforward, but relies on suitable controls, as does the isotope dilution method. The natural abundance method makes use of the observation that the nitrogenase enzyme discriminates between the nitrogen isotopes, favouring ^14^N over ^15^N. Thus, nitrogen‐fixing systems are enriched in ^14^N. It should be emphasized that all methods and thus all estimates are prone to numerous errors. Phillips (1980) further collected data from literature concerning the community efficiency of symbiotic N_2_ fixation by temperate legumes in the field. The results varied depending on cropping system and methods used, and ranged from 52 to over 300 kg N_2_ fixed per ha and year, comparable to the figures (55–140 kg/ha/year) reported by Hardy and Havelka (1975) for corresponding systems and also reported by Herridge *et al. *(2008). The annual production of industrial nitrogen is 30 Tg, whilst the nitrogen that can be fixed naturally by diazotrophic prokaryotes amounts to 100–122 Tg per year (Herridge *et al.*, 2008).

Hardy and Havelka (1975) suggested that energy was the main factor limiting N_2_ fixation in legumes. The nitrogenase system can break the very strong triple bond in the dinitrogen molecule. The reaction is energy demanding, as is the industrial reduction by the Haber‐Bosch method, which requires 400–450°C and a pressure of 200 atm to proceed (https://www.chemguide.co.uk/physical/equilibria/haber.html). Seefeldt *et al. *(2009) estimated that at least 8 moles of electrons and the hydrolysis of 16 moles of ATP are required to reduce one mole of dinitrogen. However, the reaction in legume root nodules is driven by solar energy and it can take place even at temperatures near freezing (Lindström, 1984a), as demonstrated by employing the acetylene reduction method. The nitrogenase reaction formula reveals that for one mole of dinitrogen reduced, one mole of hydrogen gas is evolved:

N_2_ + 8e^−^ + 8 H^+^ + 16 ATP → 2NH_3_ + H_2_ + 16 ADP + 16P_i_ (Hoffman *et al.*, 2014).

The evolution of hydrogen gas from soybean nodules in the absence of N_2_ was already observed by Hoch *et al. *(1957), suggesting that H_2_ evolution in the absence of N_2_ could be used to measure nitrogenase activity. If acetylene is added, nitrogenase prefers this substrate over N_2_ and at 5% acetylene, N_2_ reduction is abandoned and replaced by acetylene reduction. Since the affinity for acetylene by nitrogenase is greater than for N_2_, all hydrogen produced in the presence of enough acetylene is used to produce ethylene (C_2_H_4_). The ethylene evolved is easy to quantify by gas chromatography, and the method has provided a handy tool for nitrogenase activity measurements. It is, however, not advisable to use the method for quantification of amounts of N_2_ fixed over time, but for activity comparisons between rhizobial strains and activity measurements in diverse field conditions it is excellent (see e.g. Lindström, 1984a, 2010,1984a, 2010). A simple conversion factor between ethylene produced and N_2_ fixed is not advisable, because the acetylene reduction method is indirect, inhibits N_2_ provision to the plant by nitrogenase, and consumes all H_2_ produced. Nevertheless, acetylene reduction meant a great leap forward for research needing nitrogenase activity measurements and for practice. Hardy *et al. *(1968) describe the development of the method and its applications in detail. Hunt and Layzell (1993) gave a list of factors to be aware of when using the method, which had then been debated for almost a decade.

## The role of hydrogen in symbiotic nitrogen fixation

The evolution of H_2_ from the nitrogenase reaction was for a long time considered as energy wasted. Schubert and Evans (1976) paid attention to the evaluation of the magnitude of energy loss in terms of efficiency of electron transfer to nitrogen via nitrogenase. They measured H_2_ evolution from nodules in air and in argon, and in addition acetylene reduction activity for 19 inoculated rhizobium–legume combinations and five actinorhizal symbionts. Based on the results, they calculated the *relative efficiency* (RE) of the nodules as 1‐ H_2_(air)/H_2_(Ar) and 1‐ H_2_(air)/C_2_H_2_. The values obtained showed some variation for the individual systems depending on the method. The greatest differences were however observed between the symbiotic combinations with cowpea and mung bean inoculated with strain *Rhizobium* 32H1 (*Bradyrhizobium* according to current classification) and legumes inoculated with other strains. The measurements with soybean were repeated with shoots exposed to light and only roots incubated in acetylene, to ensure optimal energy charge for the symbiosis. Cowpea and the non‐legumes *Alnus rubra, Purshia tridentata, Elaeagnus angustifolia, Caenothus velutinus* and *Myrica californica* had superior REs, indicating that these plants have mechanisms for dealing with H_2_ loss. The REs of the other symbiotic reactions were generally around 0.50 (0.20–0.69), whereas that of cowpea was 0.99 and of 32H1‐inoculated mung bean was 0.73–0.82. Since the REs below 1.0 imply energy loss, the study prompted a renewed interest in hydrogenases in nodules.

According to the brief review by Dixon (1978), already in 1941, Phelps and Wilson (1941) reported the presence of hydrogenase in pea root nodules induced by *Rhizobium leguminosarum* ONA 311. This could not be confirmed, but the report on H_2_ evolution by soybean nodules led to investigations of pea nodules. The discovery that pea nodules did not evolve hydrogen was followed by the finding that hydrogenase was located in pea bacteroids formed by strain ONA 311. Dixon (1978) described several properties of rhizobial uptake hydrogenase and speculated about its role, suggesting that it can recycle electrons and in respiration contribute to the energy supply to nitrogenase. Further studies on RE in different rhizobial strains and symbioses showed that hydrogenases are widespread but their efficiencies vary, being one component of the RE of legume root nodules. Dr Ruiz‐Argueso contributed to work on rhizobial hydrogenases already early on (e.g. Emerich *et al.*, 1979) and went on with his group to study hydrogenases of *R. leguminosarum* (e.g. Leyva *et al.*, 1987)*.* They were able to unravel regulation of the *hup* operon by NifA protein (Brito *et al.*, 1997) and detected the presence of at least 17 common genes (*hupSLCDFGHIJKhypABFCDEX*) arranged in at least three operons with conserved gene composition and organization (Baginsky *et al.*, 2002). These *hup* genes were also identified in species of *Rhizobium*, *Bradyrhizobium* and *Azorhizobium.* However, whole‐genome sequencing of the model strain *Rlv* UPM791 revealed that the presence of *hup* genes is rather strain specific and that the genes might have different evolutionary origin in different species.

Schubert *et al. *(1978) studied the effect of hydrogenase possessing (Hup^+^) and non‐possessing (Hup^−^) strains in symbiosis with soybean and cowpea. They concluded that the Hup^+^ strains produced more biomass than the Hup^−^ ones, probably due to hydrogen recycling. The experiments were performed in axenic culture in the greenhouse. Later studies have shown that hydrogen excreted from legume nodules might serve as an energy source for rhizosphere‐dwelling bacteria (Dong *et al.*, 2003), which was already speculated about by Schubert and Evans (1976). Thus, experiments in axenic culture cannot be directly extrapolated to field conditions.

It is still not understood why nitrogenase wastes 25% of the energy obtained from ATP in the reaction, as recently noted by Hoffman *et al. *(2014) in their extensive review. The role of the excreted H_2_ has, however, been clarified in some symbiotic systems. La Favre and Focht (1983) studied the rhizosphere of *Cajanus cajan* inoculated with rhizobia possessing varying degrees of Hup^+^ activity. They concluded that all H_2_ emitted from the nodules would, independent of the Hup status, be consumed by rhizosphere bacteria within a 3‐4.5 cm radius of the nodule surface. Uratsu *et al. *(1982) collected soybean nodules from the major soybean growing areas and concluded that a majority (> 75%) of the *Rhizobium japonicum* strains isolated from those areas were lacking H_2_ uptake activity. Dong *et al. *(2003) found that pre‐treatment of soil with H_2_ increased yields of non‐legume crops and uninoculated soybean over controls, giving an indirect explanation to traditional knowledge of pre‐crop effects of legumes and benefits of legume intercropping. During the succeeding decades, the role of plant growth‐promoting bacteria in the rhizosphere of legumes and other crops has been detected (for a review see Glick, 2012). It is close at hand to conclude that the H_2_ emitted from nodules serves as an energy source for those. Recently, Li *et al. *(2018) reported that hydrogen treatment of rhizosphere soil samples of *Medicago sativa* plots influenced the composition of microbial communities as determined by amplicon sequencing of 16S rRNA genes, in comparison with untreated soil. Taken together, the role of uptake of hydrogenases for effectiveness in rhizobia remains obscure.

Hydrogen gas plays a major role in the nodule during SNF. Hunt and Layzell (1993) put great emphasis on techniques used to measure gas exchange and nitrogenase activity in nodules, especially hydrogen and oxygen, and reviewed results from several studies. Their article offers a thorough view on multiple aspects of gas exchange and is still an excellent source of information on the topic. Though H_2_ production theoretically accounts for 25% of the electron flux to nitrogenase, in practice only 40%–70% is used for SNF. H_2_ inhibition of nitrogenase might thus be an important factor in reducing nitrogenase efficiency and legume yields.

## Energy supply to nitrogenase: the O_2_ paradox

Rhizobia are obligate aerobes, thus needing oxygen for their energy metabolism, also in SNF. On the other hand, both nitrogenase proteins and the protein‐free FeMo‐Co are sensitive to oxygen with the sensitivity in the order FeMo‐co»>Fe protein» MoFe protein (Mortenson and Thorneley, 1979). The protection of nitrogenase is mediated by several mechanisms. Vance and Heichel (1991) summarized the importance of oxygen and carbon for nodule physiology and uncover several factors/processes with importance for efficiency. The nodule inner cortex, which surrounds the central zone of infected cells, serves as a diffusion barrier which limits the flux of O_2_ to the bacteroids. Together with leghaemoglobin, this barrier regulates the flux of O_2_ to nitrogenase. Further, they list plant redirection of glycolysis to malate with subsequent reductive formation of succinate under microaerobic conditions and bacteroid ATP formation coupled to a high‐O_2_‐affinity terminal oxidase as factors regulating the O_2_ flux.

Leghaemoglobin is invariably associated with nitrogen‐fixing nodules in legumes and has been used as an index of fixation potential (Virtanen *et al.*, 1955). This is due to the pigment being present in fairly constant cellular concentration within the nitrogen‐fixing cells of nodules. Its nodular concentration is therefore an index of the amount of nitrogen‐fixing tissue which is present (Bergersen, 1961). Appleby (1984), being a pioneer in nodule physiological research, summarized knowledge on leghaemoglobin and rhizobial respiration accumulated thus far, confirming the role of leghaemoglobin as a facilitator of O_2_ flux to the vigorously respiring, phosphorylating, N_2_‐fixing rhizobium bacteroids, albeit at a stabilized O_2_ tension (10 nm in soybean nodules). However, not until 2005 was it demonstrated that leghaemoglobins are required for efficient nitrogenase activity. Plant reverse genetics employing RNAi was then used by Ott *et al. *(2005) in the model legume *Lotus japonicus* to create plants carrying nodules abolished in leghaemoglobin synthesis, causing an increase in nodule free oxygen, loss of nitrogenase and absence of SNF, whereas growth on combined nitrogen was not affected. The non‐legume *Parasponia* seemed to be the only exception to N_2_‐fixing nodules having leghaemoglobin, but van Velzen *et al. *(2018) in a comprehensive study of this plant showed that this non‐legume has indeed recruited the class I leghaemoglobin HB1 for balancing oxygen levels in the nodules, whereas legumes and the actinorhizal *Casuarina* use class 2 haemoglobins for this purpose.

Following the demonstration of the bacteroid as the nitrogen‐fixing organelle (Bergersen and Turner, 1967), the energy provision for nitrogenase has been a long‐standing and complicated issue that has received the attention of scientists over the years (e.g. Bergersen, 1971; Appleby, 1984; Kaminski *et al.*, 1996; Marchal and Vanderleyden, 2000). The respiratory machineries in different rhizobia are diverse and complex. Fischer (1994) gives a thorough account of *nif* and *fix* genes in *S. meliloti*, *B. japonicum* and *A. caulinodans* identified by then, showing gene arrangements and regulatory networks. Genes, mutations in which caused loss of N_2_ fixation, were named *fix* genes. Important *fix* genes are the microaerobically induced *fixNOQP*, which encode a membrane‐bound cytochrome oxidase. *fixGHIS* genes, which map immediately downstream of the *fixNOQP* operon, encode the symbiotically essential *cbb*3‐type haem‐copper oxidase complex (Preisig *et al.*, 1996). The *fixABCX* operon, also required for nitrogenase activity, was later shown to encode an electron‐transferring‐flavoprotein (ETF) dehydrogenase (*fixABC*), whereas FixX shows similarity to ferredoxins. Delgado *et al. *(1998) reviewed genes involved in the formation and assembly of rhizobial cytochromes and their role in symbiotic nitrogen fixation. However, still many features of rhizobial respiration are unknown. The genome of *B. japonicum* encodes as many as eight different terminal oxidases for respiration in oxic conditions (Youard *et al.*, 2015). Thus, even though respiration is crucial for efficiency, no definite answers to whether respiratory activity or nitrogenase function limits efficiency of N_2_ fixation have so far been identified in this complex system.

**Figure 2:**
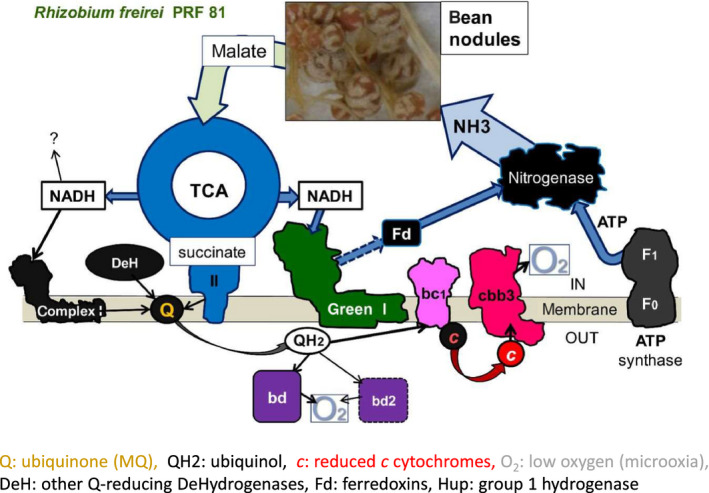
Deduced energy metabolism in nodulating symbionts of *Rhizobium freirei* and hypothetical role of green complex. Reprinted from Degli Esposti and Martinez Romero (2016) with the permission of Oxford University Press.

Modern molecular approaches now enable genome comparisons and phylogenetic approaches to be employed for renewed studies on symbiotic processes with a wider scope. Degli Esposti and Martinez Romero (2016) present an interesting advance regarding the respiratory chain of symbiotic rhizobia. They depict a current view on rhizobial energy provision to nitrogenase (Fig. 2). The respiratory complex I is a NADH: ubiquinone oxidoreductase (Nuo), and one of the largest membrane protein assemblies known. Complex I has a central role in energy production in mitochondria. Whereas the mitochondrial complex I consists of 45 subunits, the prokaryotic enzyme consists of 14 ‘core’ subunits, conserved from bacteria to humans (Efremov *et al.*, 2010; Degli Esposti, 2015). Degli Esposti and Martinez Romero (2016) compiled data from literature into a figure with enzyme expression or protein data of 16 enzymes (nitrogenase and respiratory) from *Frankia* and seven rhizobial species representing five genera. They proposed that in rhizobia the NUO14 complex 1 was downregulated in symbiosis in comparison with non‐symbiotic conditions, and the so‐called green complex I, an ancient form of the enzyme, was upregulated in *Rhizobium* and *Sinorhizobium*, but absent from the other genera. They also studied the evolution of respiratory complex I which showed that about 70 *Alphaproteobacteria*, predominantly *Rhizobiaceae*, possessed two different operons for respiratory complex I, namely the standard NUO14 and another called the green complex I (Degli Esposti and Martinez Romero, 2016). The peculiar rearrangement of gene order of green complex I was discovered in about half of the *Rhizobium* and *Sinorhizobium* strains studied by Degli Esposti and Martinez Romero (2016). The complex was always upregulated in the symbiosis of strains that possessed it. The authors thus proposed that the function of the hypothetical green complex I is to increase the efficiency of N_2 _fixation in symbiotic nodules.

## Supply of carbon and other elements

**Figure 3:**
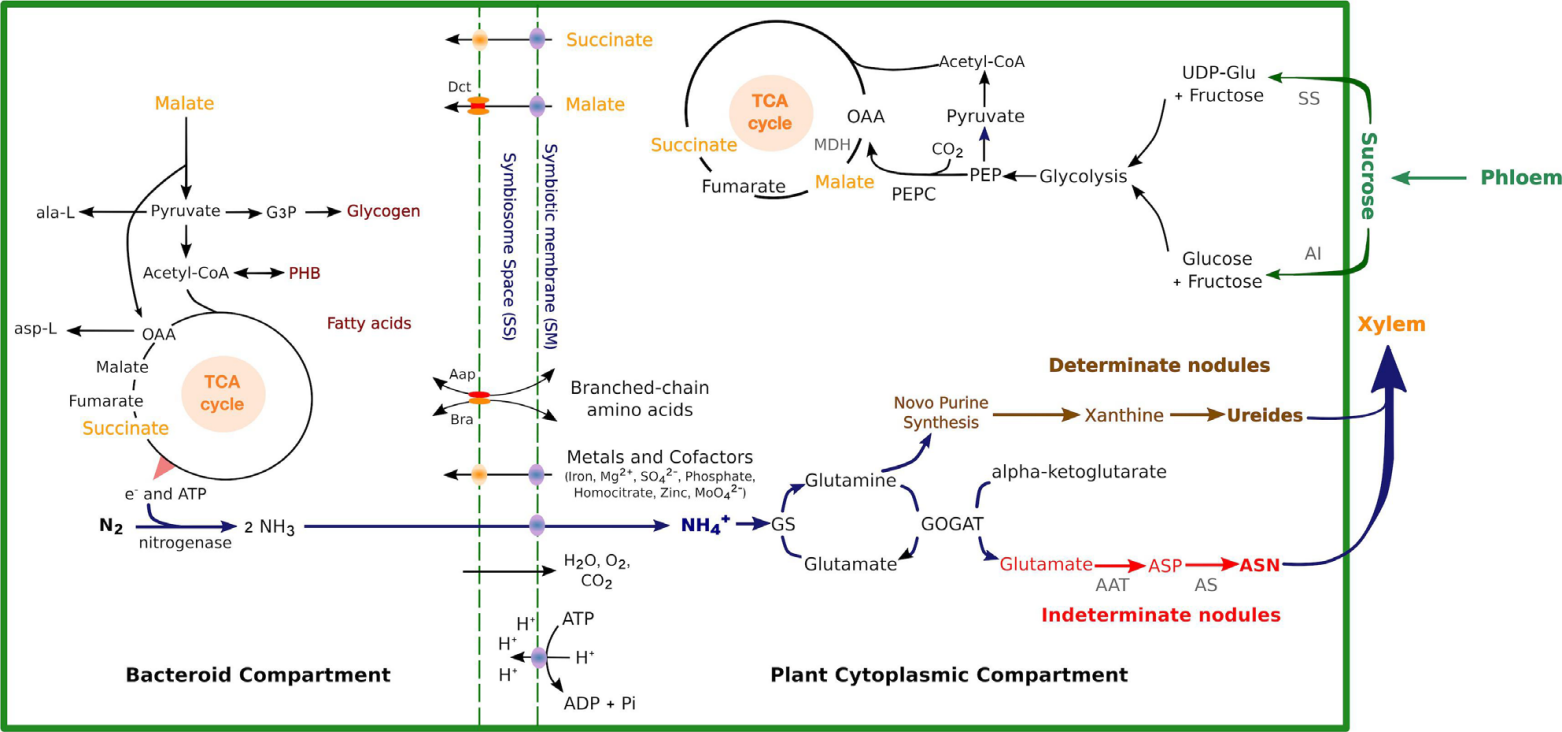
Schematics of carbon and nitrogen metabolic pathways with key enzymes, metabolites and transporters in determinate nodules and indeterminate nodules. Reprinted from Liu *et al*. (2018) with the permission of Frontiers.

Transport and metabolism of an infected nodule cell are depicted in Fig. 3 (Liu *et al.*, 2018). Since the energy used for SNF is provided by the plant, delivery of photosynthate is crucial for the SNF process. Sucrose from the shoots is cleaved by sucrose synthase in the plant cytoplasm, and hexoses are formed and further metabolized to malate. Sucrose metabolism influences the efficiency of SNF, as sucrose synthase was proven to be essential for SNF in pea nodules, demonstrated in a plant mutant, unable to form effective nodules (Gordon *et al.*, 1999). By transcriptomics it was shown in the model plant *Lotus japonicus* that several metabolic pathways appeared to be coordinately upregulated in nodules, among them are glycolysis, CO_2_ fixation, amino acid biosynthesis, purine, haem and redox metabolism (Colebatch *et al.*, 2004).

Ronson *et al. *(1981) showed that a mutant of *R. leguminosarum* sv. trifolii defective in dicarboxylate was Fix^−^ on its white clover host. Now we know that it is malate that is transported across the symbiosome membrane and further to the bacteroid by a dicarboxylic acid transporter, DctA. The gene *dctA* is regulated by *dctB/dctC,* which is one of the first sensor‐regulator systems to be characterized (Ronson *et al.*, 1984, 1987). Malate is further processed for the TCA cycle, which is central for energy and carbon provision to nitrogenase. Malic enzyme alone or in conjunction with PEP carboxykinase in combination with pyruvate kinase converts malate to pyruvate, needed for acetyl‐CoA, which condensed with oxaloacetate to feed the TCA cycle. Since the rhizobial bacteroid is sometimes limited in carbon skeletons for biosynthesis, gluconeogenesis might also occur in bacteroids (Udvardi and Poole, 2013).

The role of storage polymers in the bacteroid, such as PHB, seems to vary depending on the plant and bacterial species. Bacteroids from determinate nodules store large amounts of PHB, whereas in indeterminate nodules it might serve as storage for excess reductant, thereby influencing efficiency. Other such compounds are glycogen or lipid or even starch (Udvardi and Poole, 2013). The photosynthetic *Bradyrhizobium* sp. ORS278 has an operating Calvin cycle (Bonaldi *et al.,* 2010) and the CO_2_‐fixing enzyme RuBisCO (Gourion *et al.*, 2011), which were both shown to be important for SNF in the *Bradyrhizobium‐Aeschynomene* system and might serve a similar purpose as the storage compound PHB.

Transport of other elements has received less attention. Liu *et al. *(2018) in their review pay attention to phosphate, concluding that phosphorous deficiency affects the carbon and nitrogen metabolism in nodules and might thus affect the efficiency of N_2_ fixation. P deficiency depends on soil and rhizosphere conditions. Vance (2001) discusses the role of legumes, N and P in agriculture and emphasizes the role of mycorrhizal symbioses for P uptake. The need for Mg, Fe and Mo in the bacteroid is obvious, but very little is known about their transport. A curiosity in most rhizobia studied, is the lack of homocitrate synthase, encoded by *nifV*, which is present in free‐living N_2_ fixers. In legume nodules, homocitrate, needed for the synthesis of the FeMo‐Co, is synthesized by the plant enzyme FEN1 and transported through the symbiosome membrane. In some bradyrhizobia nodulating *Aeschynomene* species, a bacterial *nifV* gene is, however, needed for effective symbiosis (Nouwen *et al.*, 2017), and a copy of this gene was detected in the genome of *Sinorhizobium americanum* (Peralta *et al.*, 2016).

## Transport of fixed N

Ammonia formed by the nitrogenase system is most probably transferred from bacteroids via simple diffusion or via unspecific protein channels through the symbiotic membrane or through channels for NH_3_ or cations or aquaporin‐like channels. Bacteroids can thus be considered ammonium‐secreting ammoniaplasts. Once in the symbiotic plant cell, ammonia is incorporated in amino acids, mainly glutamine and asparagine, whereas in soybean fixed ammonia is transported in ureides (Udvardi and Poole, 2013). Already Herridge (1982) devised a method for estimation of SNF in ureide transporting plants, by measuring the relative abundance of ureides and nitrate in plant tissues. Amino acids present in bacteroids were for a long time confusing scientists. Their role is now thought to be provision of compounds needed before the onset of SNF and transport systems for branched amino acids have been identified for them, but they are still under study (Udvardi and Poole, 2013).

Unkovich and Layzell in 2016 (http://www.ini2016.com/1360) speculated that the nitrogenase enzyme might be N_2_ limited. Tegeder and Perchlik (2018) stress the importance of amino acid and ureide transporters localized to critical positions, where they control nitrogen partitioning, which influences nitrogen use efficiency (NUE) in peas and soybeans. Nitrogen translocation to seeds in pulse legumes contributes to seed yield, which is an estimate of the effectiveness of nitrogen fixation in pulse legumes. Optimal source–sink relationships should be achieved by matching rhizobial and plant germplasm with prevailing environmental conditions. Lindström (1984b) reported for red clover inoculated with different rhizobial strains that symbioses in which acetylene reduction activity and plant growth rate were well correlated gave the best yields. Strains aggressive at early stages of plant development might have allocated excessive resources for SNF, at the cost of other plant functions. Considering the environmental impact of crops, NUE is an important breeding and cultivation target, since it includes N yields but also losses from soil by leaching and emissions of nitrous oxide (Zhang *et al.*, 2015).

## Regulation of N_2_ fixation

NifA was demonstrated to act as a positive regulator of *nif* genes in *Klebsiella pneumoniae* around 1980 (Buchanan‐Wollaston *et al.*, 1981), and NifL was found to be a repressor. The *nifLA* operon was found to respond to combined N and O_2_. The S. *meliloti* NifA was identified by Szeto *et al. *(1984), and experiments by Ditta *et al. *(1987) with *S. meliloti* indicated that reduced oxygen tension but not combined N was potentially a major trigger for symbiotic activation of nitrogen fixation in *Rhizobium* species. Fischer (1994) in his review compared the *nif*–*fix* operons of *S. meliloti* (Rm)*, B. japonicum* (Bj) and *A. caulinodans* (Ac). In contrast to *Klebsiella*, most rhizobia studied have the *fixLJ* oxygen‐responsive two‐component regulatory system, which is involved in positive control of *fixK* (Rm, Bj, Ac) and *nifA* (Rm). FixK and FixK_2_ are positive regulators of *fixNOQP* (Rm, Bj, Ac), *nifA* (Ac), *rpoN_1_* and ‘nitrate respiration’(Bj), and also a negative regulator of *nifA* and *fixK* (Rm).

The regulatory cascades around the master regulator NifA depicted in the paper by Fischer (1994) are revisited and refined by Terpolilli *et al. *(2012) and extended to *Rhizobium etli, R. leguminosarum* and *R. phaseoli*. *B. japonicum* displays some unique features: *fixK_2_* in microaerobic conditions controls *fixNOPQ* and *fixGHIS*, genes involved in haeme biosynthesis, denitrification genes, *phbC*, *rpoN* and *fixK_1_*. *NifA* is autoregulated but also dependent on *regRS*. The three *R. leguminosarum* strains studied have different regulatory mechanisms, one strain (VF39) possessing a hybrid regulatory sensor *fixL,* for example, autoregulates *nifA,* contrary to the other strains*.* Likewise, two bean‐nodulating strains now classified as *R. etli* and *R. phaseoli* show different regulatory networks. It is obvious that diverse regulatory patterns have evolved even among strains representing the same species and even symbiovar. Based on these studies, one can conclude that several variations on the theme of regulation have evolved. This diversity might tell us something about adaptation.

## NCR peptides

A new era in BNF research was opened up with the discovery of the NCR (nodule‐specific cystein‐rich) peptides in nodules of *Medicago truncatula* (Mergaert *et al.*, 2003). By transcriptome analysis, over 300 members of this peptide family were discovered. They were characterized by a signal peptide and a conserved cysteine motif, but otherwise showed great divergence. Interestingly, they were discovered only in galegoid legumes, now renamed IRLC (inverted repeat‐lacking clade) (Wojciechowski *et al.*, 2004). Since their discovery, reports about the biological functions of NCR peptides and their occurrence have been frequent, as illustrated by several reviews (e.g. Kondorosi *et al.*, 2013; Pan and Wang, 2017; Kereszt *et al.*, 2018).

The NCR peptides are part of a larger group of peptides with biological functions involved in a wide range of processes, loosely defined as gene‐encoded small proteins of about 2 to 100 amino acids (Kereszt *et al.*, 2018). They are very diverse, illustrated, e.g. by their very different isoelectrical points. The cationic NCR peptides seem to have the greatest influence in nodules. The nodule development in IRLC legumes is special in that bacteroid maturation inside the indeterminate nodules of legumes from this clade shows remarkable similarity to host cell differentiation. Since the effects of NCR peptides resemble antibacterial peptides (AMPs) or plant defensins, causing cell death, Van de Velde *et al. *(2010) investigated the role of these peptides in the morphological changes of rhizobia observed in IRLC legumes. Using *M. truncatula* as a model, they found that the NCR peptides indeed were involved in the terminal differentiation (endoreduplication, cell enlargement, loss of cell division capacity, and increased membrane permeability) in IRLC legumes. These bacteroids are not able to convert to a free‐living state, whereas bacteria inside determinate nodules with no differentiation can revert back to a free‐living state. *In vitro*, NCR peptides display antimicrobial activity against a broad range of bacterial species; however, only a few NCR peptides have been studied in detail and the precise *in planta* activity of NCR peptides remains uncertain.

The process of terminal differentiation, repeated DNA replication (endoreduplication) without cell divisions, results in extensive amplification of the entire bacterial genome and elongation of bacteria, revealing a positive correlation between DNA content and cell size, similar to that in eukaryotes (Mergaert *et al.*, 2006). This endoreduplication results in up to a 128‐fold increase in cell DNA. By comparing different legumes, Montiel *et al. *(2016, 2017) were able to describe different nodule morphotypes: (S) standing for swollen bacteroids, found in five out of six major papilionid subclades; (EB) displaying elongated and branched bacteroids, in *Medicago, Vicia* and *Galega* belonging to the vicioid subclade; (E) bacteroids meaning elongated; (SP) standing for spherical bacteroids; and (U) for undifferentiated ones. A correlation between the number of NCR peptides and bacteroid morphology was found in all the studied IRLC legumes, including the Vicioid *Cicer, Ononis, Trifolium* and *Pisum*, in addition to *Medicago, Vicia* and *Galega,* as well as in Astragalean *Astragalus*, Hedysaroid *Onobrychis* and *Glycyrrhiza*. The highest number of NCR peptides predicted from nodule transcriptomes was around 600 in *Medicago truncatula*, whereas in *Glycyrrhiza uralensis* only seven were detected. EB bacteroids were characteristic of plants with high NCR numbers. NCR peptides or their genes were so far found only in plants forming indeterminate nodules, and were absent from at least *Lotus japonicus, Glycine max, Glycine sojae, Phaseolus vulgaris* and *Cajanus cajan* with U‐type bacteroids. The terminal differentiation of bacteroids has, however, only been properly studied in the IRLC legumes and in *Aeschynomene* in the Dalbergoid clade, in which NCR‐like peptides have been detected (Czernic *et al.*, 2015; Montiel *et al.*, 2017). In the Dalbergoid *Aeschynomene*, bacteroids are in some species of the elongated type, whereas in other species in this genus and in the likewise Dalbergoid *Arachis hypogaea* the bacteroids are large spheres (SP type), just like those of the IRLC *Cicer arietinum and Ononis spinosa.* It has been assumed that the host determines the bacteroid and nodule types (e.g. Bakkou, 2011). However, Crespo‐Rivas *et al. *(2016) showed that *Sinorhizobium fredii* HH103 bacteroids were not terminally differentiated in the nodules of the IRLC legume *Glycyrrhiza uralensis,* an exception from the terminally differentiated nodules harbouring *Mesorhizobium tianshanense* HAMBI 3372, as observed by Montiel *et al., *(2016, 2017). Lamouche *et al. *(2019b) reported a relationship between bacteroid type and effectiveness. A transcriptomic analysis on E‐ and S‐type bacteroids formed by *Aeschynomene afraspera* and *A. indica* nodules identified bacterial functions activated in bacteroids specific to each bacteroid type. More studies are needed to further investigate the role of bacterial strains for this process.

Kereszt *et al. *(2018) give an update on the current knowledge of plant peptides in rhizobium–legume symbioses. Already in response to Nod factors, plant peptides are involved in processes leading to nodule formation and SNF. Two systemic regulation processes are especially prominent. Peptides of the CEP family exert a positive effect on nodulation in response to N starvation, whereas CLE is a negative regulator of nodule numbers. Other peptides contribute to nodule formation. The RALF and DLV peptides negatively affect the infection process in early stages of nodule development. During later stages, RALF has a negative but DLV a positive effect. ENOD40 and miPEP172c affect primordium formation, uORFp1 participates in the control of meristem maintenance, and apart from NCR peptides, GRP and SNARP peptides probably control bacteroid differentiation and functioning. All of these have their own roles in nodule formation and SNF. The NCR peptides, however, are more relevant for the topic of this minireview.

The cationic NCR247 is involved in a complex interaction network, which affects bacterial cell division machinery by binding to FtsZ, preventing septum formation, and also translation, and protein folding (Farkas *et al.*, 2014). Pan and Wang (2017) reviewed recent developments regarding molecular events guiding bacteroid development in indeterminate nodules. These nodules can be divided into 4‐5 zones, as first described by Vasse *et al. *(1990): nodule meristem, infection zone, interzone, fixation zone, and senescence zone. Determinate nodules lack a persistent apical meristem and do not possess the respective zones, just the fixation zone*.* NCR peptides all have in common that the signal peptide is cleaved by the DNF1 signal peptidase complex, ensuring excretion of the peptides into the bacteroid (Wang *et al.*, 2010). According to their working model, thioredoxin s1 acts on disulfide bridges of cysteines producing oxidized peptides, mainly in the infection zone. NCR peptides NCR211 and NCR169 were identified as essential for N_2_ fixation (Horvath *et al.*, 2015; Kim *et al.*, 2015), after two ineffective plant mutants *dnf4* and *dnf7* were found to be defective in genes encoding these peptides. NCR211 acts in the infection zone and the interzone, NCR169 mainly in the interzone and fixation zone (Pan and Wang, 2017).

Rhizobia can defend themselves by producing the HrrP metallopeptidase, expressed in all nodule zones in bacteria carrying the *hrrP* gene (Price *et al.*, 2015). Two accessions of *Medicago truncatula*, A20 and A17, were compared. Strain *S. meliloti* B800 was Fix^+^ on A17 but Fix^−^ on A20. Fix^+^ was restored on A20 by curing strain B800 of a 199‐kb plasmid. Further studies demonstrated that this plasmid carried the *hrrP* gene. HrrP expression caused the rhizobia to escape terminal differentiation and contributed to maintain living cells inside the nodule. It seems that the NCR peptides contribute to the host specificity of nitrogen fixation and that their role is to optimize nitrogen supply to the plant by specifically stimulating bacteroids to grow and fix N_2_ and not become parasitic, the so‐called ‘cheaters’ (Oono *et al.*, 2009; Van de Velde *et al.*, 2010).

NCR peptides can also be involved in the selection of the bacterial partner, since incompatibility between *M. truncatula* ecotype Jemalong and *S. meliloti* strains Rm41 and A145, which form effective symbioses with other *Medicago* partners, is eliminated from nodules by allelic forms of two peptides, NFS1 and NFS2 (Wang *et al.*, 2017). In IRLC legumes, especially in the vicioid clade, the symbiotic interaction is especially fine‐tuned. It has thus been further speculated that NCR peptides contribute to efficiency of N_2_ fixation by stimulating bacteroids to grow, prolong the lifetime of nodules, and reduce senescence in the senescence zone, especially in strains lacking HrrP. Oono and Denison*et al.* (2010) give an overview of the phylogeny of 40 Papillionid species with nodule and bacteroid types indicated. The tree can guide in the selection of symbioses for comparative studies. It shows that swollen bacteroids have multiple origins and might confer some host benefits, such as optimization of N_2_ fixation efficiency, as further elaborated by Lamouche *et al. *(2019b) for *Aeschynomenae* species.

The rhizobial *bacA* gene*,* encoding an ABC transporter*,* was identified in *Sinorhizobium meliloti* by Glazebrook *et al.* (1993) as being essential for bacteroid development. Its role was further manifested by Haag *et al. *(2011) as critical for the legume symbiosis, by protecting *S. meliloti* against the bactericidal effects of NCR AMPs. In *Bradyrhizobium,* a corresponding gene *bclA* was identified as being necessary for bacteroid differentiation in *Aeschynomene.* Since the NCR peptides in IRLC and Dalbergoid legumes seem to have evolved independently, phylogenetically diverse legumes seem to have evolved convergently. A productive N_2_‐fixing symbiosis can be seen as the result of a carefully maintained balance between the effect of NCR peptides and the ability of rhizobia to resist them. The result of this interplay might contribute to host specificity.

## NO in symbioses

Nitric oxide (NO) is a gaseous reactive oxygen species (ROS) that has evolved as a gaseous intracellular and intercellular signalling molecule. NO has a diverse range of regulatory functions (signalling hormone) in many physiological processes in mammals. Beside animals, NO functions as a signalling molecule involved in plant growth and development, as well as in plant response to abiotic and biotic stresses (Boscari *et al.*, 2013a; Domingos *et al.*, 2015). In plants, NO can induce the plant defence system, and thus evolution of pathogens armed them with the mechanism of NO detection and modulation and adaptive response to this signal (Meilhoc *et al.*, 2010). NO per se, at high concentration, can become very toxic, and Meilhoc *et al. *(2010) showed that the presence of NO in culture could inhibit the growth of *Sinorhizobium meliloti*. Regarding SNF, NO can potentially act as a nitrogenase inhibitor (Boscari *et al.*, 2013b). Cueto *et al. *(1996), however, reported a putative NO synthase in lupine nodules, by which arginine is converted to NO. In 1998, Mathieu *et al*. demonstrated that a complex of NO and leghaemoglobin (Lb) was detected in the nodules formed by *Bradyrhizobium japonicum* strain Bj110 in *Glycine max*. In the 2000s, production of NO was observed in bacteroid‐containing cells of nodules of *Medicago truncatula* and *M. sativa* and in mature nodules of *Lotus japonicus* (Baudouin *et al.*, 2006; Pii *et al.*, 2007; Shimoda *et al.*, 2009). Since 2010, many hypotheses were proposed to explain the sources of NO and the effects of NO on the molecular dialogue between legumes and rhizobia, some of which are presented here (for reviews, see Meilhoc *et al.*, 2011; Boscari *et al.*, 2013a; Hichri *et al.*, 2016).

The origin of NO and the responses of plant and rhizobia to NO at different steps of the SNF is diverse. The production of NO in the early stage of rhizobium–legume interaction can occur as a result of plant‐defence activity (Boscari *et al.*, 2013a). Studying the SNF interaction between *Mesorhizobium loti* and *Lotus japonicus* showed an accumulation of NO in the root surface in the first four hours after inoculation (Nagata *et al.*, 2008). The lipopolysaccharide (LPS) molecules produced by *M. loti* induced NO production in the roots of *L. japonicus* (Murakami *et al.*, 2011). In mature nodules, both plant and bacteroid partners are involved in the production of NO (Hichri *et al.*, 2016). The studied sources of NO and NO scavengers in SNF interactions are listed in a recent article by Berger *et al. *(2018). In summary, the specific NO‐generating systems in SNF interactions can be listed as follows: the mitochondrial electron transport chain, nitrate reductase, nitric oxide synthase and nitrate‐independent nitrate reductase in the plant, and in the bacteria nitrate reductase, periplasmic nitrate reductase (Nap) and nitric oxide synthase (Berger *et al.*, 2018). In nodules, the nitric oxide synthase (NOS)‐like NO generating system (O_2_ consuming process) occurs in normoxic tissues, while in hypoxic or microoxic conditions, nitrate reductase (NR) and mitochondrial electron transport chain (ETc) mostly occur (Meakin *et al.*, 2007; Sánchez *et al.,* 2010; Horchani *et al.*, 2011). Performance of transcriptomic experiments in the past two decades identified thousands of plant genes (Ferrarini *et al.*, 2008; Meilhoc *et al.*, 2011; Boscari *et al.*, 2013b; Hichri *et al.*, 2016) and hundreds of rhizobial genes (Hyduke *et al.*, 2007; Meilhoc *et al.*, 2010; Hichri *et al.*, 2016) as upregulated by NO. Ferrarini *et al. *(2008) showed that among 999 genes upregulated as a response to NO in roots of *M. truncatula*, 290 (278 induced and 12 repressed) genes were detected during nodule development. This finding supported the hypothesis of involvement of NO in nodule development. Furthermore, NO upregulates various genes participating in flavonoid biosynthesis (e.g. chalcone synthase), development of root hairs at the nodulation stage (kinases, receptor‐like kinases, transcription factors), in carbon metabolism (sucrose transport, sucrose synthase, isocitrate dehydrogenase, glyceraldehyde‐3‐phosphate dehydrogenase or malate dehydrogenase), in nitrogen metabolism (glutamine), and in proteasome‐dependent proteolysis (Meilhoc *et al.*, 2011). Moreover, NO in concert with reactive oxygen species (ROS) are key regulatory players of the redox reactions of symbiosis (Puppo *et al.*, 2013). Shimoda *et al. *(2005) demonstrated that NO upregulates the expression of *LjHb1* that encodes nsHb1 protein. The expression of Hb1 modulates the level of NO (downregulating) to reduce plant defence response and permitting reception of symbiont in the roots (Murakami *et al.*, 2011). The other regulatory role of NO on nodule development occurs via plant genes involved in nodule development, such as *MtCRE1* and *MtCCS52A* (Ferrarini *et al.*, 2008; del Giudice *et al.*, 2011).

**Figure 4:**
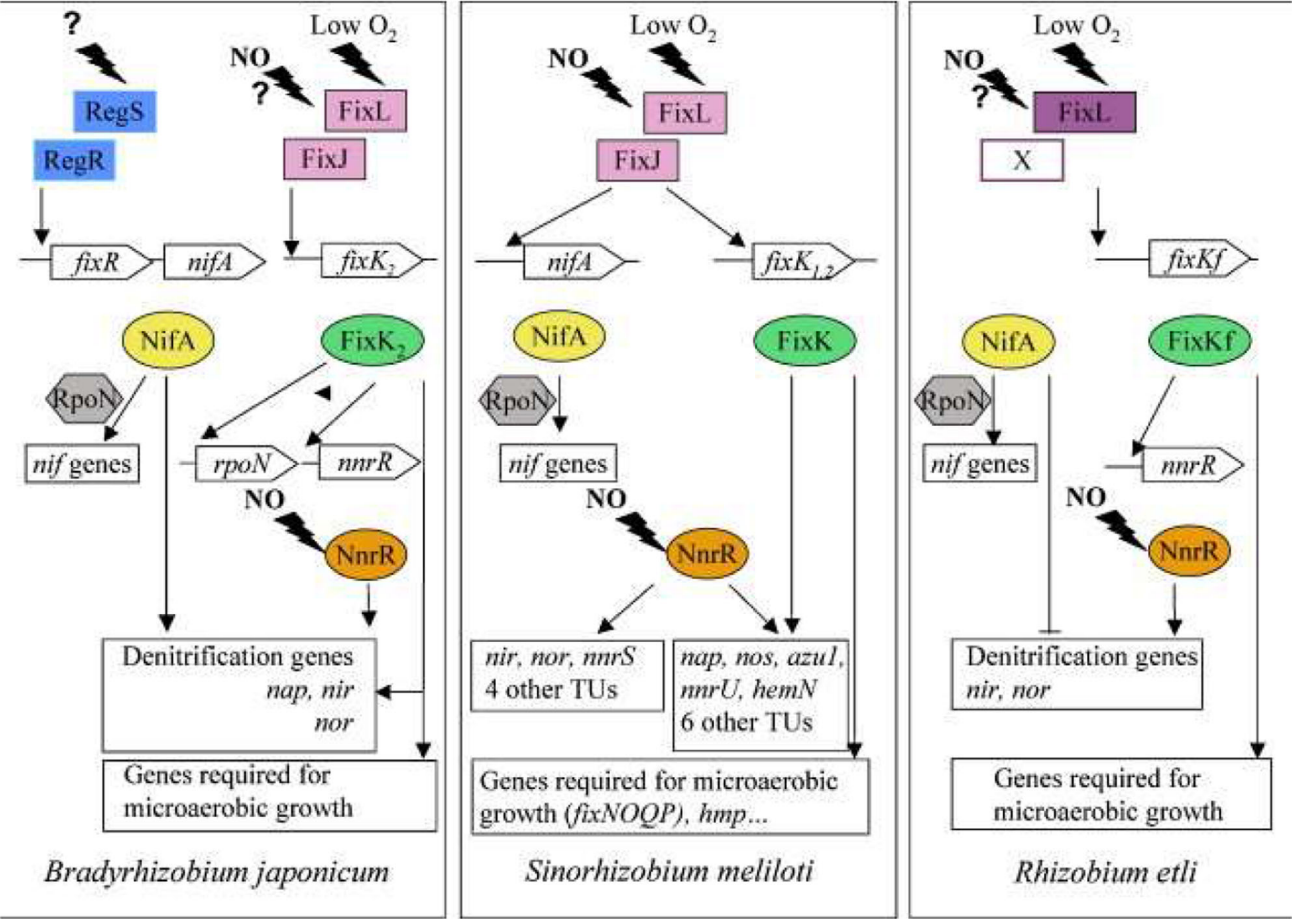
Comparison of NO signalling cascades in the symbiotic bacteria *Bradyrhizobium japonicum, Sinorhizobium meliloti* and *Rhizobium etli*. TU: transcription unit. Reprinted from Meilhoc et al. (2011) with the permission of Elsevier.

The other scenario of response to NO, a toxic molecule for bacteria, occurs in rhizobia (Fig. 4). The majority of rhizobial genes upregulated in response to NO are regulated through either FixLJ or NnrR (Meilhoc *et al.*, 2010). The signal of low oxygen leads to autophosphorylation of FixL (hem‐based sensor kinase) that transfers the phosphoryl group to FixJ (response regulator). Phosphorylated FixJ triggers the transcription of FixK and NifA genes (Tuckerman *et al*, 2001; Meilhoc *et al.*, 2011). NnrR, a member of FNR/CRP family, is a NO‐dedicated sensor. Tosques *et al. *(1996) reported that the products of NnrR were responsible for production (by nitrate reductase, *nir*) and reduction (by NO reductase, *nor*) of NO in a denitrifying strain of *Rhodobacter sphaeroides*. NnrR could be considered as the regulator that can tune the level of nitrate and NO in nodules, and protect nitrogenase against inhibition by these molecules. Furthermore, flavohaemoglobin (Hmp) and two proteins of the NnrS family (NnrS1 and NnrS2) are involved in NO detoxification (Meilhoc *et al.*, 2010; Cam *et al.*, 2012; Blanquet *et al.*, 2015; Hichri *et al.*, 2016). Interestingly, in one of our studies (Österman *et al.*, 2015), we found that the superior *Neorhizobium galegae* nitrogen fixers harboured *nnrRS* genes that could not be identified among the other strains.

## A diversity and evolutionary perspective

Enormous development regarding the symbiotic interactions has been made during the last decades. *Medicago truncatula* with its microsymbiont *Sinorhizobium meliloti* has been used as a model system for symbioses with indeterminate nodules induced, whereas *Lotus pedunculatus*– *Mesorhizobium loti* has served as the model for symbioses with determinate nodules. Great advances were also made concerning rhizobium phylogeny and taxonomy. However, including both the bacterial and the legume symbionts in experiments rapidly increases the amount of experiments needed, which thus increases and becomes difficult to handle. Looking for determinants governing symbiotic efficiency becomes like looking for a needle in a haystack. Traditional screening for effective symbioses, i.e. inoculation of selected rhizobia on plants, can reveal superior combinations useful in agriculture. But the mechanisms behind the results are seldom revealed.

The recent studies on the role of NCR peptides*, bacA* and *hrrP* for symbiosis, referred to above, evoked a renewed interest in molecular mechanisms behind efficient symbioses and host specificity. Comparisons of N_2_ fixation in diverse hosts producing different bacteroid types showed that bacteroid morphology makes a difference. Peas and peanuts with EB and SP bacteroids, respectively, had greater fixation efficiency than beans and cowpeas with U bacteroids inoculated with the same rhizobial strain (Oono and Denison, 2010). Investigating bacteroid differentiation may reveal mechanisms behind these observations, useful for further research into the topic of swollen bacteroids. Are they symbiotically more efficient than non‐swollen ones, and can we this way modify other host species to have higher nitrogen fixation efficiency as well?

Price *et al. *(2015) screened 150 *Sinorhizobium* isolates and noted that about 20% of natural isolates harboured *hrrP* homologs, all of which appeared to be encoded on large accessory plasmids. Further, diCenza *et al. *(2017) in their study on definition of a minimum rhizobium symbiotic genome, using the model *S. meliloti–M. sativa* symbiosis, performed studies with *S. meliloti, S. fredii* and *R. leguminosarum* to explore the role of *bacA* for specificity. Using *bacA* protein sequences from 82 sequenced genomes, they found that the S. *meliloti* BacA has evolved towards a specific interaction with *Medicago,* more related to some *Gammaproteobacteria* than to those of other rhizobia*.* They concluded that their results highlight the limitations of using a single model system for the study of complex biological topics.

Matching compatible bacterial and plant accessions for improved performance is important. Our own work with *N. galegae* and its host plants *Galega orientalis* and *G. officinalis* has shown that symbiotic bacteria and host plants co‐evolved (Suominen *et al.*, 2001). The bacterial species *N. galegae* can be divided into two symbiovars, orientalis and officinalis. Both symbiovars nodulate both host plant species, but depending on with which host plant an effective symbiosis is formed, we distinguish the symbiovars. Other legume species are not nodulated by these bacteria. By studying a collection of bacterial root nodule isolates representing either symbiovar or plant corresponding accessions from the Caucasus, we demonstrated that the diversity of *N. galegae* sv. orientalis bacteria as well their host *G. orientalis* was greatest in their gene centre Caucasus, in comparison with sv. officinalis and its host, which are thought to originate in eastern Mediterranean (Österman *et al.*, 2011).

A comparison of ten whole‐genome‐sequenced *N. galegae* strains, five of each symbiovar, displayed similarities but also interesting differences. Both symbiovars possessed a *nifQ* gene, demonstrated to be indispensable for N_2_ fixation by gene displacement. However, the *N. galegae nifQ* was very divergent from model gene sequences from *Klebsiella pneumoniae, Azotobacter vinelandii, Rhizobium tropici* and *Sinorhizobium fredii*. All sv. orientalis strains tested carried an additional sigma factor gene, *rpoN_2_,* which was shown to be necessary for effective N_2_ fixation on *G. orientalis*. New genes that were identified as specific for strains of one symbiovar may be involved in determining host specificity. Many additional genes involved in transcriptional regulation or in metabolic functions of strains with similar growth promoting properties were identified and might play a role in the fine‐tuning of effectiveness (Österman *et al.*, 2014, 2015).

Li *et al. *(2012) investigated 159 endophytic bacteria isolated from surface‐sterilized root nodules of wild perennial *Glycyrrhiza* legumes growing on 40 sites in central and northwestern China. Amplified fragment length polymorphism (AFLP) genomic fingerprinting and sequencing of partial 16S rRNA genes revealed that the collection mainly consisted of *Mesorhizobium, Rhizobium, Sinorhizobium, Agrobacterium* and *Paenibacillus* species. Based on symbiotic properties with the legume hosts *Glycyrrhiza uralensis* and *Glycyrrhiza glabra*, the nodulating species were divided into true and sporadic symbionts. Five distinct *Mesorhizobium* groups represented true symbionts of the host plants, the majority of strains inducing N_2_‐fixing nodules. Sporadic symbionts consisted of either species with infrequent occurrence or species with weak or no N_2_ fixation ability.

The studies by Degli Esposti and Martinez Romero (2016) point in a similar direction. The studies of respiratory complex I revealed a hypothetical role for this complex and provide new perspectives also to understanding the physiological and biochemical links between the respiratory chain and N_2_ fixation in rhizobia. The green complex I operon has been lost in several nodulating organisms but retained in rhizobia showing superior N_2_ fixation capacity. Possibly, the function of green complex I increases the efficiency of N_2_ fixation in the nodules.

## Future perspectives – can we define effectiveness?

One aim of SNF research is the sustainable production of food and feed. For optimal production, efficient symbioses are desirable. To ensure nodulation and N_2_ fixation, farmers inoculate legume seed with bacterial preparations. Selection of inoculant strains is mainly done from bacterial collections created by isolation of strains from host nodules often sampled in diverse environments. Strains are first tested for efficacy in controlled environments and selected bacteria are taken to the field. An important property of the inoculant strains is their competitiveness, but there is no general rule for selection of competitive strains other than plant testing. An ideal strain should perform well and be competitive in the respective environment, but not be too persistent. This issue has been known for decades and is thoroughly reviewed by, e.g. Vlassak *et al. *(1997). Martínez *et al. *(2016) argued that rhizobial strains are continuously evolving in plants and therefore recommend an approach inspired on experimental evolution studies where strains adapted to particular conditions may be selected. The selection and detection of efficient rhizobial strains should take place under local field conditions in order to obtain superior nitrogen‐fixing symbionts.

Can we then make use of recent findings to improve yields? Terpolilli *et al. *(2012) present a comprehensive list of metabolic mutations which alter symbiotic N_2_ fixation. Can that information be useful? The vast knowledge of gas exchange, regulation, respiration, NCR peptides and so on has brought an insight into intricate mechanisms which do not per se reveal an answer to the big question of efficiency, but help us understand the systems.

One reason for the boost of research into SNF in the 1970s was the wish to replace fossil energy with solar power for the production of combined N. This is still a very good, though often neglected, reason to boost symbiotic legume use in agriculture. Steffen *et al. *(2015) in their paper on planetary boundaries regarding several human‐induced disturbances on globally critical functions estimated regarded reactive N to be close to its planetary boundary. By growing N_2_‐fixing legumes, one can contribute to soil conservation and health and to sustainable food production. N_2_O release from agricultural land contributes to global warming. Rhizobial inoculants with complete sets of denitrification genes might in the future mitigate N_2_O emissions from legume fields (Mania *et al.*, 2019).

We have reviewed some of the putative bottlenecks and listed old and new discoveries, many at the molecular level. However, it might be wise to pay more attention to the diversity of natural systems and look at how SNF has evolved to cope with constraints and yet survived to be productive in varying environments. Vance and Heichel (1991) wrote that the evolution of exquisite adaptations that facilitate the N_2_ fixation process is extremely efficient in biological terms. Udvardi and Poole (2013) concluded that knowledge of how transport and metabolism are integrated to achieve effective SNF and how they vary in different symbioses may help us to identify bottlenecks in SNF in specific legume–rhizobia systems. Degli Esposti and Martinez Romero (2016) put the attention to the respiratory chain, and recent discoveries regarding NCR peptides add to the factors to be taken into account. The regulatory roles of NO during symbiosis reveal yet one interesting player in this field.

Plant endophytes and bacteria inhabiting the rhizosphere have been reported to enhance nodule formation and tolerance of biotic and abiotic stress in controlled conditions (e.g. Egamberdieva *et al.*, 2010, 2013, 2016, 2017a, 2017b,2017a, 2017b). These plant growth‐promoting rhizobacteria (PGPR) represent diverse taxa and have sometimes been successfully used as biofertilizers. Hydrogen excreted from N_2_‐fixing root nodules might help feed the PGPR (Schuler and Conrad, 1991). Co‐inoculation with *Azospirillum* + *Bradyrhizobium* on soybean seed was evaluated by Hungria *et al. *(2015), who found beneficial plant effects under different soil and climate conditions in Brazil. For practical purposes, carefully selected and tested, high‐quality plant growth‐promoting biofertilizer formulations could replace synthetic N fertilizer applied to legumes worldwide. By locally adapting crop rotations and mixed cropping, more sustainable food production could be achieved in the near future. This is the immediate aim of SNF in the near future.

## Conflict of interest

There is no conflict of interest.
